# Understanding and using patient experiences as evidence in healthcare priority setting

**DOI:** 10.1186/s12962-019-0188-1

**Published:** 2019-09-23

**Authors:** Leah Rand, Michael Dunn, Ingrid Slade, Sheela Upadhyaya, Mark Sheehan

**Affiliations:** 1 Board on Health Sciences Policy, National Academies of Sciences, Engineering, and Medicine, 500 Fifth Street NW, Washington, DC 20009 USA; 2 Ethox Centre, Nuffield Department of Population Health, Big Data Institute, La Ka Shing Centre for Health Information and Discovery, University of Oxford, Old Road Campus, Oxford, OX3 7LF UK; 3 Highly Specialised Technology Program, Centre for Health Technology Evaluation, National Institute for Health and Care Excellence, 10 Spring Gardens, London, SW1A 2BU UK; 4 Wellcome Centre for Ethics and Humanities, University of Oxford, Oxford, UK

**Keywords:** Resource allocation, Patient involvement, Public involvement, Patient experience, Decision-making, Priority setting

## Abstract

**Background:**

In many countries, committees make priority-setting decisions in order to control healthcare costs. These decisions take into account relevant criteria, including clinical effectiveness, cost-effectiveness, and need, and are supported by evidence usually drawn from clinical and economic studies. These sources of evidence do not include the specific perspective and information that patients can provide about the condition and treatment.

**Methods:**

Drawing on arguments from political philosophy and ethics that are the ethical basis for many priority-setting bodies, the authors argue that criteria like need and its effects on patients and caregivers are best supported by evidence generated from patients’ experiences. Social sciences and mixed-methods research support the generation and collection of robust evidence.

**Results:**

Patient experience is required for a decision-making process that considers all relevant evidence. For fair priority-setting, decision-makers should consider relevant evidence and reasons, so patient experience evidence should not be ignored. Patient experience must be gathered in a way that generates high quality and methodologically rigorous evidence. Established quantitative and qualitative methods can assure that evidence is systematic, adherent to quality standards, and valid. Patient, like clinical, evidence should be subject to a transparent review process.

**Discussion:**

Considering all relevant evidence gives each person an equal opportunity at having their treatment funded. Patient experience gives context to the clinical evidence and also directly informs our understanding of the nature of the condition and its effects, including patients’ needs, how to meet them, and the burden of illness. Such evidence also serves to contextualise reported effects of the treatment. The requirement to include patient experience as evidence has important policy implications for bodies that make priority-setting decisions since it proposes that new types of evidence reviews are commissioned and considered.

## Background

Healthcare costs have continued to rise, and healthcare systems increasingly rely on gatekeepers or regulators to control costs. In some countries, these take the form of explicit committees, like the UK’s National Institute for Health and Care Excellence (NICE), Germany’s Institute for Quality and efficiency in Health Care (IQWiG), or the Swedish Council on Health Technology Assessment (SBU). Elsewhere, payers make decisions, like US insurance companies determinations of the coverage offered, or local boards that allocate a health budget. Each “committee” of decision makers does or ought to take into account relevant criteria such as clinical effectiveness, cost-effectiveness, and patients’ need [1–4]. Whether these criteria give a committee reason to fund treatment depends on the evidence for each. Much of the evidence is drawn from clinical, including epidemiologic, and economic studies. However, criteria such as the nature of the condition and its effect on the lives of patients as well as the effect of the treatment on their lives can only be properly supported by evidence generated from patients’ experiences of the condition and the treatment.

In this paper, we will argue that patient experience should play a role *as evidence* in healthcare decision-making. In the course of making this argument we distinguish this role for patient experience from other kinds of patient or public involvement. Typically, these do not seek to generate systematic evidence of the kind used in evaluating the criteria for a health technology assessment.

Consideration of patient experience as evidence is an important element of the decision-making process for priority setting: its consideration alongside other forms of evidence helps to ensure equality of opportunity by making certain important evidence is not overlooked. Patient experience, considered as evidence, contributes to understanding the nature of the condition, the effect of the treatment, and the effect of the treatment on patients’ and carers’ lives, which is all evidence relevant to the decision. It will therefore be important that not only decision makers but also patients understand their role in contributing to the evidence for a decision.

In response to likely concerns about the quality and standards of patient evidence, we suggest research methods that might be used to generate the evidence. Treating patient experience as evidence, in the way we suggest here, uses systematic methodology rather than personal anecdote to generate evidence that can be rigorously considered alongside scientific evidence. Concerns about quality can be met with good methodologies, and should not pose a barrier to using patient evidence in decision-making for priority setting.

## Methods: A role for patient experience as evidence

It seems fair that patients who will be directly affected by the healthcare committee’s decisions are in some way included or involved in the decision-making process.^1^

This statement seems to capture something important about the justification of a role for patients in decision-making. The immediate question is how this thought survives reflection on the nature and justification of the decision-making process. For example, it is unclear whether (i) this is about involving patients in the decision, as decision-makers for example or (ii) about the value and role of their experience as relevant evidence for the decision [7, 8].

In what follows we consider the second of the alternatives mentioned: the focus of this paper is to consider the ways in which patient experience can and should act as evidence in the deliberations of healthcare limit-setting or commissioning committees. In subsequent sections, we develop a robust account of the nature of patient experience as evidence and what counts as high quality evidence of this type.

Following on from the thought expressed above, we may think patients have a right to be heard—inclusiveness in these processes means allowing patients or patient groups to express their views and have their say [9–13]. Here we do not question this right or any of the ways in which it may be articulated. The process through which difficult healthcare resource allocation decisions are made should explicitly allow all stakeholders, especially patients and patient groups, the opportunity to a fair hearing and should actively promote the involvement of a wide range of patients and patient groups in the decision.

These issues of process, however, are crucially distinct from the content of the decision to be made and the obligation on the decision-maker to ensure that the decision-making process gathers all of the relevant evidence and makes its decisions in light of all of that evidence. Evidence is an issue of content, not process. This paper is concerned with evidence as decision-making content and will not directly consider the very important questions concerning how best and most appropriately to involve patients and patient groups in the decision process.^2^

This also means that the paper focuses squarely on the nature of the evidence that is required for the decision and ways of ensuring that the evidence is of the highest quality. Therefore, we set aside questions about the right to be heard, the right to have a say and the ways in which these rights might be protected.

## Results: What are the arguments for integrating patient experience as evidence into a healthcare priority-setting committee’s decision-making?

### 1. Why should patient/carer experience be treated as evidence?

This is the key question. On the face of it, the experience that patients have in living with a particular condition and with its (attempted) treatments is important in understanding: the nature of the condition; the impact it has on the lives of those with the condition and those around them; the potential for treatments to assist with the condition and the impact it has on life; and the reality of the impact of new treatments for the condition [19–25]. The experience of patients in these areas is a crucial resource for decision-makers on priority-setting committees if they are to properly understand the condition and the treatment.

Beyond the instrumental value of patient experience, the literature has not engaged with the questions of why and how these experiences should feature in the process *as evidence* alongside the other forms of evidence, such as that from scientific and economic analyses. This section lays out the justification for its inclusion.

The claim that patient experience should be gathered and presented as part of the evidence considered in the decision-making process depends on the arguments about the nature of the decision and the justification of the process. The decision-making process should be established in such a way that the best (and fairest) decisions can be made in difficult circumstances.

One might assume that the fairest way to distribute scarce resources is to hold a lottery since this gives each person an equal chance at receiving scarce resources. While each person is equally likely to have her needs met, it will quickly become apparent that the lottery does not meet other substantive values that are widely held: it does not account for how sick a person is, i.e. how great her need is, it does not consider how effective the treatments are or how much each one costs, some may even object that it does not consider who wins the lottery and that some features, like age, matter. The lottery thought experiment shows that there are substantive considerations, other values, that matter in addition to the fairness of the process by which winners and losers are chosen. However, while there may be general agreement on the sorts of substantive values that matter (need, effectiveness, etc.) there is not agreement on *how much* each one matters. The pragmatic approach to the theoretical positions of wanting both an equal process and taking into account disagreement on substantive principles is to propose a process that treats participants fairly but also allows for deliberation and debate on how to weight different kinds of values. Four key features of this argument justify a process that includes equal consideration and the use of evidence, including patient evidence, are as follows:

i. There is reasonable disagreement about what is the right decision for funding a treatment in a range of cases. This feature of the resource allocation context provides the grounding for the process: it is in the light of this disagreement that the process can be considered to be a fair method of making a decision.

The initial problem of making just decisions about healthcare resources—the problem of allocating these resources in the fairest way—is noticeably defined by a distinct kind of disagreement: people can reasonably disagree about what the right course of action is [26–30]. Some people will judge that the right decision is to fund a particular treatment because those who are likely to benefit from it are in desperate need of help. Others will think that the costs, combined with doubts about the likelihood of benefit, are such that resources should not be allocated to this treatment.

This disagreement is reasonable because it can and does occur between individuals who satisfy a number of important characteristics. Reasonable disagreement is disagreement between individuals who (i) understand that a decision needs to be made, (ii) understand that others are likely to disagree, (iii) understand that there are a range of relevant criteria to be considered and to which others may appeal in the defence of their view, (iv) are able to accept that others may value the criteria in different ways for a given decision and (v) recognise that the processes involved in coming to a decision centre on reasons and reasoning.

The problem of making decisions when there is reasonable disagreement is pervasive in modern, democratic societies that recognize that citizens will hold different views of what is morally right, and this is often the case for making determinations about shared or public goods, like healthcare. If people cannot agree on the right way to distribute healthcare resources, then a fair process leads to decisions that follow a procedural standard rather than applying a specific moral, substantive view to each one.

ii. All people should be given an equal opportunity at having the treatments for their condition being funded, which is achieved by following a fair process and giving equal consideration of relevant criteria from available evidence in the decision-making process.

Because people can reasonably disagree about the way in which healthcare resources should be allocated and so whether particular treatments should be funded, a process which enables all parties to have a fair hearing for their cause is one plausible system for helping to ensure that justice is done. “Equal opportunity” here means that each person or patient group has equal access to the fair process, equal opportunity to present their case and evidence, and the case should be judged on its particular merits and on the basis of all the available evidence. Each case equally follows the process even if the same outcome does not result. This is different from a lottery that gives each person an equal chance, which raises other concerns discussed above, and instead emphasizes the procedural consideration of substantive values. Equal opportunity is connected to the idea, familiar from the work of John Rawls, of justice as fairness [31]. It also underpins the accountability for reasonableness model develop by Daniels and Sabin [27, 28]. Whilst there may be other systems for ensuring justice in resource allocation, this is the one that has been adopted in the literature and by several healthcare committees, such as those in the UK. In our view it is also the most robust and defensible [26, 31–35].

An ‘equal opportunity at having their treatment funded’ is a claim about the content of the decision-making and how the decision-making process is constructed. It means that the various stakeholders’ perspectives and reasoning should be included in the decision-making process. This must include patients and carers with the condition in question and also those with an interest in the treatment or who will be responsible for its delivery. Importantly, in each case, an equal opportunity refers to what evidence and reasons are considered as a part of the decision-making process and how they are considered.

iii. Equal consideration and so equal opportunity requires that all relevant reasons are considered fully and in the light of all of the relevant evidence.

Giving and considering reasons for and against funding is the key element of the fair process [27–29, 36–38]. A decision is made reasonable by the way it is justified, i.e. the reasons that are given for it. Part of this justification will depend on the evidence that supports the reason for a decision. An articulation of why a decision has been made should include reference to the evidence that supports that reason. This justification may also refer to other considerations and evidence that were not strong enough to support the alternative decision. A process like this is similar to a judge hearing a case: if the judge were pre-decided on the facts and outcome, then the fair legal process would be upended. A fair process relies on the openness of reasoning and consideration of all available evidence. Courts limit what evidence is admissible, which is similar to the concept of considering relevant, appropriate evidence, including patient experience as evidence: in Part B, we discuss the standards for patient evidence and suggest the methodological rigour for it such that it can be included in decision making.

Ultimately there are only two sources of genuine disagreement within a system that is characterised by reasonable disagreement.^3^ First, people may disagree about the value of a particular consideration—about how much weight it should have in this particular case. So, individuals may disagree about how important cost-effectiveness is in a particular case, particularly when compared to patients’ need. Second, people may disagree about the strength of the evidence in support of a particular consideration—about the degree to which the evidence shows that a consideration is in play. So individuals can disagree about whether we have good evidence of patients’ need or of the clinical effectiveness of a treatment. Both will clearly affect the reasoning behind the decision and the decision itself.^4^

Both of these sources of disagreement are intractable in this context. The process is not designed to resolve this problem but to enable a fair decision in the context of it.

iv. If all relevant reasons for and against a particular funding decision are to be considered fully and fairly, a full range of evidence in support of those reasons must be also considered.

Patient and carer experience of the nature of the condition, its effects on their lives, and the effects of the treatment is relevant to the decision-making and so should be formally included within it. This experience, like all other forms of evidence, should feature in the process as evidence in support of the criteria used in the process. Just as health economic evidence about costs and clinical effectiveness can support reasoning about cost-effectiveness, so too, evidence about what it is like to live with a condition can support reasoning about need: the nature of the condition, the effect of the condition on lives of sufferers and their carers, and the way in which the treatment might play a role in their lives. In short, this experience can be important evidence for decision-making and, given our interest for reasons of fairness in the inclusion of the full range of evidence, the experiences of patients and carers should be included.

### 2. How should patient/carer experiences as evidence, function in decision-making?

It is one thing to justify that these experiences ought to be a part of the decision-making process as evidence but it is another to give an account of how, in general terms, this evidence might play a role in those decisions.

The patient and public involvement literature is replete with examples of the way information provided by patients can bring context and depth to other sources of data collected in clinical research studies. Such context and depth is seen to provide a fuller account of these alternative data sets and to assist in maximising the relevance of such data sets when translated from ‘bench to bedside’. However, this literature invariably fails to make reference either to the role that this added value might play in the actual processes of decision-making or how patient experiences more generally (and not about clinical research) should feature in funding decisions [7]. In developing the argument in the section above, we have relied on the idea that there is a range of relevant criteria that should be taken into consideration in the process of making fair resource allocation decisions. These criteria represent the range of ethically relevant reasons that decision-makers may have for deciding to fund or not fund a particular treatment [27–29, 36, 38]. For example, clinical effectiveness clearly matters in the decision about funding; if a treatment had no effect, this would arguably give decision-makers an overwhelming reason not to fund it [39]. Cost-effectiveness, as a measure of value for money, is another criterion that we can readily understand to be a relevant reason for or against funding. If a treatment is not cost-effective, i.e. not good value for money, it is reasonable not to fund. Finally, it is easy to see that patient need is a criterion that is relevant to the decision: if patients are not in need, say because the symptom of the condition ameliorated by the treatment is of little concern, there would be little reason to fund the treatment. Of course in cases where the patients’ need is extreme, this need provides a very strong reason to fund a treatment that promises to meet it.

These three examples illustrate the way in which the criteria function to provide reasons for particular decisions. Clearly in actual cases the criteria and so the reasons pull in different directions: perhaps most often, patient need gives decision-makers reason to fund but cost-effectiveness provides a reason not to fund. The process described above requires that each of the criteria are treated and considered equally. Most importantly, it means that decision-makers should not approach the decision-making task with a prejudged value attached to any of the criteria. They should not be predisposed to value one more highly than another: to do so would be to fail to treat all stakeholders equally in the decision-making process. We assume the equal stance of stakeholders since these decisions occur in the context of allocating healthcare resources to those who are participants in the system, either as insurance-premium or tax payers.

Evidence functions in a definite way to provide support for reasons. So evidence of patient need provides a reason to fund a treatment whereas evidence of a lack of need provides a reason not to fund. Similarly, evidence of clinical effectiveness provides a reason to fund whereas evidence of no effect provides a reason not to fund. In each of these cases the evidence does not necessarily provide an over-riding reason—what gives us the strongest or an over-riding reason will depend on the other reasons, their relative strength, and the judgement of the decision-maker. Also, clearly, lack of evidence gives no reason either way.^5^

So far the argument has shown that fairness requires equal opportunity and that equal opportunity requires equal consideration of relevant criteria. Equal consideration of relevant criteria requires attention to the full range of reasons for and against funding a treatment. Proper attention to all these reasons is possible only when adequate evidence is provided in support of each.

In the examples above, we have mentioned only patient need, clinical effectiveness and cost-effectiveness as relevant reasons. As we will see below, there are a number of others that should be included. We will also discuss how the decision-making process and criteria used function as a range of relevant reasons that ought to be considered.

#### Which kinds of criteria are those for which patient experience can provide evidence?

When making decisions about new treatments, committees use criteria such as the nature of the condition, the impact of the new treatment, the cost and cost-effectiveness of the treatment, and the treatment’s impact beyond direct health benefits.^6^

The idea is that each of these criteria is to be considered by the committee in order to make a decision about funding. In this way, these criteria function as the relevant considerations that play a central role in making the process of decision-making a fair one—it is because these criteria capture the possible range of relevant reasons that the process is able to be fair and procedurally just. Following from the arguments above then, evidence which supports each of these criteria is also crucial in making the process a fair one.

It is important, therefore, to articulate clearly how accounts of patient and carer experience connect to the decision criteria. There are three ways in which patient and carer experience can function as evidence in support of these criteria—in each case the experience of patients and carers is importantly placed to support or weaken the role the criteria play in the decision-making reasoning. Three of the six criteria are most relevant here: (a) the nature of the condition, (b) the impact of the treatment, and (c) the impact of the treatment beyond direct health benefits. The other criteria depend heavily on knowledge of the costs associated with providing the treatment. Patients and carers are not well placed to contribute to these criteria.

- The nature of the condition and its effects on the lives of patients and their carers.

As we have suggested, patients and carers can provide important evidence about the needs that they experience living with the condition [21, 41, 42]. The evidence that they can provide about the course of their lives gives substance to the decision-makers’ understanding of the nature of the condition. This criterion can then be supported in reasoning about the decision: ‘Because we now know more about what it is like to live with this condition, we can see more clearly the nature of the need that these patients have.’ More specifically, patient evidence can provide accounts of:

i. the lives of patients, family and carers.
ii. what is of value in those respective lives.
iii. the perceived lack/deficiencies in the lives of patients, family and carers.
iv. the ways in which these deficiencies could be met (articulated in terms that do not rely directly on the effectiveness of the treatment).

In each case the evidence provided is able to support reasoning about the needs of patients and carers, which informs a decision.

b. Patients’ and carers’ experience of the condition and its effects on their lives means they can contextualise the clinical evidence that is provided to the committee.

Patients’ and carers’ knowledge and experience of the condition enable them to provide comment on the significance and relevance of the outcome measures used in research, the relevance of the research to the lives of patients with the condition, and the relevance of both clinical research and quality of life assessments. In this way patients’ and carers’ experiences provide evidence about the value of the research that has been conducted on the treatment under consideration [22, 43–50]. Patients’ and carers’ experience of the condition and its effects on their lives means they can contextualise the purported effects of the treatment.

An account of the experience of the treatment and its affects on the lives of patients and carers adds important context to the findings of clinical research [22, 46, 47, 50]. These are properly understood as ‘raw’ experiences that do not make claims attributing causal responsibility but which can sit alongside clinical effectiveness data. Qualitative evidence from patient experience cannot prove effectiveness, but it can give context and inform feasibility and acceptability of clinical research. For example, in a case where there is strong evidence of clinical effect, patients’ experience of the treatment may strengthen the value of that evidence by adding qualitative evidence around the effects documented in the research. Conversely, in the same case, patients’ experience may undermine actual value of the demonstrated clinical effect.

## Discussion: What does good quality patient evidence look like?

In the previous section, the nature and value of understanding patient experience as evidence has been articulated, and the role that such evidence ought to play in decision-making clarified in light of the relevant kinds of criteria.

While these arguments clarify why patient evidence ought to be used, there are plausible concerns about the quality and methods of gathering patient experiences together as evidence. These concerns need to be addressed: if decision-makers are not confident of the quality of the evidence presented, the purpose of using patient evidence will be undermined. Moreover, if such evidence is of poor quality there is a danger that decisions made by the decision-makers that are contingent on this evidence will lack an adequate justification. For any kind of evidence, poor quality evidence should not be used to inform decisions. Therefore, it is important to address concerns about patient evidence and demonstrate that it can be systematically collected and evaluated.

### 1. Concerns about the quality of patient evidence

#### i. The systematic generation of patient evidence

The first set of concerns focuses on ascertaining whether patient evidence has been obtained systematically. These worries highlight the need for clarification about the overall methodological framework guiding how patient evidence is collected, analysed and presented.

In order for this evidence to be treated as being of equivalent status to other forms of evidence assessed in the process, the framework within which the evidence was generated must be appropriate. If this can be guaranteed, then the risk of bias in patient evidence would be reduced and concerns that it is a marketing ploy, mere anecdote, or a single case study that lacks external validity with limited force as evidence generalised to the patient group as a whole.

#### ii. The adherence to quality standards in generating patient evidence

The second set of concerns focuses on the adherence to particular quality standards in the systematic production of the patient evidence. Patient evidence must avoid the incorporation of bias and subjective interpretation. In order to meet evidence standards, it should be generated by researchers using rigorous methods and with appropriate knowledge in this field.

Adherence to certain quality standards is crucial to ensure that the patient evidence presented at the committee meetings can be taken to be a *robust* and *valid* account of patients’ experiences. Without such standards, decision-makers are likely to be sceptical about the evidence and dismiss it from serious consideration in their evaluations, as they should with poor quality evidence.

The concern about quality standards has been raised in the literature on evidence of this kind, and a number of solutions have been proposed by social scientists working in the area of health research [51–53]. Because healthcare decision-making committee members are often healthcare professionals or service providers, they may be most familiar with biological, epidemiological, or economics research traditions but not with methodologies that have different standards. Explaining the methodologies of social inquiry and the way the evidence is produced to the decision-makers will be important to ensuring its proper consideration.

Below, we suggest how patient evidence could be generated in light of research standards articulated within social scientific inquiry. We show how these standards can shape (i) how studies are designed in order to document patient experiences in a methodologically systematic way that is tailored to the requirements of the committee’s evaluation, and (ii) how specific quality considerations are upheld in the process of collecting and analysing this kind of evidence.

### 2. Articulating a methodological framework for generating patient evidence

For data about patient and carer experiences to be counted as valid research evidence it should be gathered in a systematic way. Therefore, it is important that those responsible for generating this evidence have a clear methodological framework within which they are operating, and that this framework is orientated directly and explicitly towards the requirements of the committee’s evaluation process. The framework that we articulate below could underpin a number of different methodologies or methods within the social sciences. The appropriateness of each methodology and method needs to be considered in light of this general framework.

#### i. Qualitative studies of patients’ experiences

Patient experience generates evidence about the nature of the condition and the impact of the technology under evaluation on the person’s health and broader quality of life. For this evidence to be most useful, it is important to capture a rich and in-depth account of patients’ lives so that all salient features about what it means live with the condition—and what it is like to receive an intervention to manage or treat this condition—are documented to the fullest extent.

In contrast to other kinds of evidence that the committees review, the depth and detail of understanding required here points towards a qualitative study design. Qualitative inquiry includes prolonged and detailed contact with a person’s life situation, aims for a ‘holistic’ overview of the context under study, and attempts to capture data ‘from the inside’ by being responsive to the participants’ insights rather than being driven solely by the researchers’ priorities [54].

Traditional social research methodologies within the domain of health aim to produce new understandings about some aspect of health care or health conditions. One commonly adopted methodology is ‘grounded theory’ [55–57], where the aim of qualitative inquiry in a healthcare setting might be to provide a robust, theoretically-rendered account of the ‘lived experiences’ of a particular health condition. In the decision-making process, however, patient experiences as evidence are of value only in so far as these experiences can substantiate the relevant criteria the healthcare committee is using. The aim is not to understand patient experience for its own sake. Therefore, inductive methodologies such as ‘grounded theory’ are unlikely to be appropriate for the process. Instead, a methodology adopted to generate patient evidence must be shaped directly by the criteria outlined in the healthcare committee’s guidelines. Otherwise, although a rich, detailed understanding of patients’ experiences will be obtained, this understanding will fail to capture experience in ways that are useful to the committee members tasked with evaluating this evidence by reference to their criteria.

This key observation implies that an appropriate methodology for generating patient evidence will need to be ‘top-down’ in nature, developing, shaping (and potentially limiting) accounts of patients’ experiences such that they can flesh out the relevant criteria that healthcare committee members are required to take into account when evaluating a specific technology.

Adopting a ‘top down’ approach to generating patient evidence has a number of practical implications for the study design and how the outcomes of the study inform the evaluation process:

- *Sampling strategy* Patients, carers and family members who are selected to share their experiences must be chosen on the grounds that (i) they have direct experience living with the condition, (ii) or that they have direct experience caring directly for those living with the condition, or (iii) that they have direct experience of the technology being evaluated (whether this be in their own country or elsewhere). This suggests a systematic though non-probabilistic sampling strategy, such as purposive sampling, should be endorsed.
- *Methods for data collection* Interviews with patients, carers and family members that aim to understand the experiences of these individuals must be structured in such a way that they focus solely on the criteria relevant to the particular healthcare decision-making process. This suggests that a structured or semi-structured interview strategy should be adopted in order to keep the elucidation of experience ‘on track’.
- *Methods for data analysis* The analysis of these interviews must be undertaken in ways that place the relevant criteria at the heart of the analytic process. For example, a ‘thematic analysis’ of patients’ experiences—where commonly occurring insights emerge in the process of coding interviews [58, 59]—must be documented within these pre-determined criteria. One well-established and highly structured approach to qualitative data analysis that would fit these requirements is ‘Framework Analysis’ [60, 61].
- *Presentation of the evidence* The evidence must be presented to the healthcare committee in a systematic way, derived explicitly from the structured analytic framework used to capture the different themes within the data and their relationship to the committee’s evaluation criteria. Extracts from interviews with patients, carers and family members should be presented as illustrative of the general insights obtained about patients’ understandings of the nature of the condition, and the experience of the treatment under consideration. They should not be presented as stand-alone examples of particular and unique personal experiences. A pro-forma approach for presenting the patient evidence to the committee would be one strong option, and such an approach would be entirely consistent with the methodological requirements and analytic process outlined above.

#### ii. Quantitative surveys of patients’ experiences

Qualitative inquiry sheds light on the range of relevant patient experiences, but gives limited insight into how these experiences are distributed across the patient group as a whole. It is possible, for example, that a range of experiences about what it is like to live with the relevant condition will be documented from the qualitative study, but that one of these experiences will prove to be common to all patients, whilst other experiences apply only to a minority of patients. Patient evidence can incorporate the extent to which different kinds of experiences are common to all patients, and that may be especially important to a healthcare committee making population-level decisions.

For this reason, we recommend a mixed-methods methodological design that incorporates a survey of experiences across the relevant patient, carer and family member groups as a whole. The development of a patient questionnaire to undertake such a survey should be a secondary component of the methodological design, and the content of the questionnaire should be shaped directly by the range of experiences captured in the qualitative study. Existing validated questionnaires to measure health outcomes are unlikely to be fit for the purpose of the committee’s role (though established questionnaire measures of this kind might be useful for obtaining standardised outcomes measures that document clinical effectiveness in other ways. In our suggested mixed-methods design, the various items of the survey must be derived directly from the key insights from the interviews (and, again, these items must correspond with the relevant criteria to be taken into account in the particular evaluation process). As the aim here is to document the commonality of the range of health experiences documented in the interviews, a questionnaire design that incorporates a Likert scale is likely to be sufficient.

It is likely that the mixed-methods approach will be more compelling in the evaluation of some technologies rather than others. When the patient group affected by any given technology is relatively small, it might well be possible to conduct interviews with most of these patients, negating the additional value of conducting a survey. In contrast, a large patient group will mean that interviews will only be able to be conducted with a small sample of these patients. In these cases, we strongly advise that a survey is conducted following the interview study. Though we recognise that the feasibility of undertaking this study design will be contingent on surveying potentially large groups of patients affected by a particular health condition.

To conclude, for patient experiences to function as evidence, it is important that these experiences are captured within a systematic methodological framework that has been designed to meet the requirements of the particular healthcare technology evaluation process. We recommend a mixed qualitative and quantitative study design that adopts certain key methodological characteristics to capture the relevant experiences in a systematic and reliable way.

### 3. Adhering to quality standards in generating patient evidence

Once a systematic framework for generating patient evidence has been articulated, we need to turn our attention towards the quality standards that should guide this process. Concerns about the ‘quality’ of social inquiry have been raised over the last two decades, particularly in the context of health services research [62, 63]. Some of these concerns arise because of the significant philosophical disagreements between social scientists about the nature of the knowledge produced by both qualitative and quantitative approaches within social research.^7^ Other concerns stem from misunderstandings by those within more experimental empirical research traditions who see qualitative research as the ‘easy option’, lacking methodological sophistication, denying the importance of statistical representativeness, incorporating problematic degrees of subjective interpretation, and suffering from small sample sizes [64]. Position statements and evidence from qualitative studies of resource allocation processes in the NHS suggest that the requirement to rationalise funding decisions might at least in part be due to committee members’ difficulty in handling patient involvement and positioning patient experience data of different kinds in committee processes and judgements [65–67].

In light of the broader methodological concerns, there have been significant attempts within the social research literature to articulate a common set of ‘quality standards’ that enable the validity and robustness of a piece of qualitative research to be judged on its own terms [68–70]. Such standards are methodological ‘way-markers’ functioning to guide the process of collecting, analysing and presenting data in ways that enhance the validity of the evidence gathered. Given the concerns about bias and subjectivity in patient evidence, endorsing these quality standards in generating patient evidence will be a key aspect of ensuring that this evidence is recognised as being fit for purpose by committee members.

There are a range of quality standards available in the literature that share some common features. One particular set of quality standards that has been extremely influential in social research is that put forward by Mays and Pope [38]; another, which is employing influential standards in qualitative evidence synthesis is CERQual [71, 72]. We present this account as an illustrative example, and seek to expand on each standard to show how these requirements have particular design implications for the different stages of the process of generating and presenting patient evidence in this context. We recommend that healthcare committees examine the different quality standards that have been articulated in the social research literature, and consider carefully which set of standards would best function to enhance the validity of patient evidence, whilst simultaneously alleviating the concerns about quality.

The six quality standards articulated by Mays and Pope [38] are:

i. *Triangulation* By comparing the outcomes of two different methods for data collection, the researcher can be confident that the evidence produced is comprehensive in its scope, and that an overall interpretation of the data has been corroborated by the different data sources available. This standard can be maintained by combining an interview phase and a survey phase in the generation of patient evidence. For example, if the survey results suggest that some patients who complete the questionnaire do not agree that the kinds of experiences captured within the items of the questionnaire are comparable with their own experiences, this would suggest that not all relevant experiences have been captured in the interview phase of the study, and that further interviews need to be undertaken.
ii. *Respondent validation* The researcher can reduce analytic error by establishing that his/her interpretation of these data corresponds to the experiences that the study participants were trying to convey in the research process. This standard applies to the analysis of the interview data. Respondent validation generally requires the use of ‘member checking’, where a number of the patients interviewed are asked to comment on whether the central themes that are captured in the process of analysis match their experiences. If the patients are not able to validate these interpretations, further analysis of the data is required. In the survey phase of the study, validation of the responses from the patients also needs to be considered.
iii. *Clear exposition of methods of data collection and analysis* The researcher needs to ensure that those reviewing or considering the data know precisely which methods were adopted, and how the decisions to use these methods were made. This standard applies to the presentation of the data, to those tasked with reviewing and critiquing the evidence, and to the committee members who need to review this evidence to evaluate the particular technology. Any critique of the evidence must be able to ‘track back’ to the decisions made by the researcher in designing the study, and a clear justification for why certain techniques or methods were employed needs to be provided for this evidence to be validated as fit for purpose.
iv. *Reflexivity* The researcher needs to be sensitive to the multiple ways in which s/he, or the research process itself, has shaped how the data has been collected and how it has been interpreted. This standard applies to the collection, analysis and presentation of the data. Being sensitive to the researcher’s own positioning in the research process requires that any biases that might have been imported (i) through the researcher’s prior assumptions, (ii) through the degree of ‘distance’ s/he has from the patients interviewed, or (iii) because of the effects of his/her personal characteristics (age, gender, professional status etc.), need to be documented and discussed carefully. Steps should be taken to ensure that such biases are reduced to the greatest possible extent. If, for example, the decision is made that a member of the patient organisation should interview other patients on the grounds that this will increase the quality of the experiences shared by the patient, careful consideration should be paid to ensure that this researcher does not impose his/her own understandings of patient experiences into the dialogue. Equally, if an independent researcher is conducting these interviews, consideration should be given to whether the professional status of this researcher might limit the experiences that the patient feels comfortable sharing, and how rapport between the researcher and the patient in the interview setting could be improved [73, 74].
v. *Attention to negative cases* The researcher can improve the validity of the evidence generated by focusing on ‘outlier’ or ‘deviant’ cases that can help to refine and refocus the analysis to gain a comprehensive picture of the object of study. This standard has implications for sampling and for data analysis. In deciding who to interview, it is important to follow up interviews with patients whose experiences appear to be contradictory to the general accounts of patients’ experiences that emerge as the interviews progress. Focusing subsequent interviews on these ‘outlier’ or ‘deviant’ cases can help to refine and refocus the analysis to gain a comprehensive picture of the range of patients’ experiences that should be taken into account in the evaluation of a technology. The requirements of data ‘saturation’ ought to be invoked and applied here. If the interviews being conducted with patients continue to give rise to different kinds of experiences that had not been captured in previous interviews, further interviews should be conducted in order to shed light on these different experiences.
vi. *Fair dealing* The researcher must ensure that a wide range of perspectives is incorporated into the study design and that the views of one group are not over-represented. Adopting a non-probabilistic sampling strategy is likely to ensure that this standard is met. Recruiting people for interviews such that the maximum variation in patient experiences is captured will act to ensure that the insights of one single person, or group, can be taken to be generalisable. Again, the strategy of ensuring data ‘saturation’ will assist in meeting this standard, as will the researcher taking steps to ensure that the views of carers and family members are captured, as well as just the experiences of the relevant patient group.

### 4. Critiquing the patient evidence

Even when these standards have been followed in the generation of patient evidence, it is not clear that healthcare committees are best placed to ascertain whether the evidence presented to them meets these methodological and quality standards (unless the committee has a qualified health services researcher skilled in mixed-methods social research methodologies applied to health). It is important, therefore, that individuals with expertise in social research methodology are able to critique the patient evidence generated, prior to it being made available to the committee.

In order to respond to valid concerns about standards and quality, there looks to be an important place for the independent appraisal of the evidence generated from patients’, carers’ and family members’ experiences. We recommend that a patient evidence review group made up of experts with knowledge and experience of social research methodologies is appointed to critique this evidence, such that the committee members can be reassured that the submissions from patient groups can be taken to be high quality evidence about patients’ relevant experiences.

Given that the methodological guidance outlined above would support a range of different methodologies and methods, it is important that any review process enables the expert reviewers to enter into a critical dialogue with those responsible for generating this evidence. The researchers should be prepared to be able to articulate and defend the methodological framework within which they have sought to generate accounts of patients’ experiences.

The patient evidence review group (or the committee member who possesses this expertise) should then report their views about the quality of this evidence to the healthcare committee. It should be clear to the committee members whether the evidence has been generated in ways that meet the standards of qualitative or mixed-methods inquiry, as it may well be the case that the committee members judge poor quality evidence to have less weight in the evaluation of the particular technology.

Once an appropriate methodological framework, a clear set of quality standards, and a transparent evidence review process has been established, the healthcare committee can be confident that the patent evidence generated is valid, robust and relevant. If so, it will be fit for purpose to be considered alongside other clinical evidence necessary to flesh out the relevant criteria in making resource allocation decisions. When committee members are trained to recognise how different methodological approaches and research standards underpin the different kinds of evidence that they are required to consider, the patient evidence generated for their consideration should be able to be incorporated into the healthcare technology evaluation.

## Conclusions

Priority-setting committees make challenging decisions about the commissioning or provision of healthcare services based on reasoning supported by the best available evidence. The first part of the paper argued that these committees have an obligation to consider relevant criteria in the decision-making process. To support these criteria, evidence provides reasons for making particular decisions. While there is a strong practice of using clinical, epidemiologic, and economic evidence for these decisions, patient experience can provide important, relevant insight into the nature of patients’ need, the condition, and the treatment under consideration. The second half of the paper describes features that should be used to guide the generation of patient evidence in order to ensure its validity.

The claim that patient evidence is necessary to support a fair process of decision-making has broad implications for all decisions made that affect patients’ access to treatment. Such decisions are regularly made by formal committees conducting health technology assessments, like those in the UK or Sweden, and by healthcare payers, such as the US insurance companies. Some of these bodies, like the UK’s NICE, have articulated processes to promote fairness in their decision-making, and patient evidence should be added to these decision-making procedures, barring the practical concerns about costs and implementation. For those healthcare committees that fail to meet the criteria for a fair process, it should be clear from the second half of the article that even they can make use of patient experience as a valuable form of evidence to inform decisions. Following through on the arguments presented in this paper requires a policy shift amongst priority-setting bodies towards commissioning evidence reviews of patient experience and including this evidence in their deliberations.

The importance of patient evidence stands out particularly when other forms of evidence are scant. Patient evidence may provide a useful evaluation tool for new healthcare technologies that have limited clinical data to support claims of effectiveness. For example, in the case of ultra-orphan conditions, it may be near impossible to run adequately powered clinical trials. Robust patient evidence, generated via the framework we have described, could then be some of the most clearly articulated evidence available regarding the new technology.

This paper has offered a new account of the need to include patient evidence in the decision-making process. This claim is not grounded in an argument about inclusiveness or a right of patients to express their views. Rather, the necessity of patient evidence in decision-making is grounded in the obligation to consider evidence relevant to the decision. Patient evidence is one such kind of evidence, and, if generated according to an appropriate methodological framework, it is essential information to the decision-making.

## Data Availability

Not applicable.

## Footnotes

1 Other patients and patient groups are not directly affected—this is related to the lack of explicit opportunity costs. This idea is very clearly present in the literature [5–7]. Notice that if the decision was between two (and only two) treatments for different patient groups, then we might reasonably require representatives from both groups to be present. But since in most (?) real cases there are either a substantial number of ‘competing’ treatments or a decision about an individual treatment in the context of an overall budget (i.e. against many and some unknown treatments), the involvement of ‘competing’ patient groups is not plausible.

2 Examples of involving patients in healthcare research and decisions, see [14–18].

3 Other sources of disagreement will be a product of a failure in reasonable disagreement: either as an unwillingness to appreciate the possible positions that others might take or a misunderstanding about the terms of the process.

4 The strength that a reason has involves the value that we attach to that reason in this case. Evidence supports reasons but does not determine the value or weight that we give to it.

5 “Lack of evidence is not evidence of lack.”.

6 These example criteria are explicitly taken from the Interim Methods Guidance for NICE’s Highly Specialised Technology Evaluation Committee, however they are familiar from similar contexts and capture the general range of considerations [3, 40].

7 Put crudely, these debates centre on a disagreement between positivist and constructionist accounts of the nature of social scientific knowledge. Positivists contend that social research produces a truthful account of an external social reality, and constructionists contend that social research is nothing more than an event within which social life is constructed, contingent on the form of the research encounter and the unique interpretations that take place within it, and denying any external social reality that is tracked in this process.
